# The Academic Background of Youth Soccer Coaches Modulates Their Behavior During Training

**DOI:** 10.3389/fpsyg.2020.582209

**Published:** 2020-09-24

**Authors:** David Agustí, Rafael Ballester, Jordi Juan-Blay, William G. Taylor, Florentino Huertas

**Affiliations:** ^1^School of Doctorate, Catholic University of Valencia, Valencia, Spain; ^2^Department of Physical Education & Sport Science, Catholic University of Valencia, Valencia, Spain; ^3^Carnegie School of Sport, Leeds Beckett University, Leeds, United Kingdom

**Keywords:** coach training, feedback, communication pattern, notational analysis, coaching

## Abstract

This investigation aims to explore the relationship between the academic backgrounds of youth soccer coaches (U10 and U12 age groups) in Spain and the type of verbal behavior used during training sessions. The sample consisted of 70 coaches divided into two groups, depending on whether or not they had engaged with a university-level academic studies related to Physical Education and or Sport Sciences. A modified version of the “*Coach Analysis and Intervention System*” (CAIS), developed by Cushion et al. (2012), was used to collect data. A total of 32,886 verbal behaviors were noted and analyzed. Our results suggest that the coaches with university academic backgrounds frequently use more verbal behaviors and that these could be associated with positive effects on the players’ learning and development processes. We suggest it is important to develop specific training programs aimed at optimizing the coaches’ communicative and socio-affective skills in order to maximize their impact in youth athletes’ learning process.

## Introduction

Communication, in its different manifestations (verbal or non-verbal), directly affects the optimization of the learning process and represents an important factor influencing positive athlete development (Allan and Côté, 2016). Coaches should use effective communication to promote positive relationships with, and among, players by developing a sport performance model focused on human development (Turner et al., 2018). The most common type of communication between coaches and athletes in sports is based on the use of verbal instructions.

Studies have shown that verbal strategies and coached-created motivational climates are particularly useful for improving sports performance and motivation in children and young athletes (Cushion and Jones, 2001; Torregrosa et al., 2008; Møllerløkken et al., 2017).

Longarela et al. (2015) contended that verbal behaviors aid the reinforcement of players’ decisions and actions, throughout the match and training conditions across the age groups. Thus, studying the coaches’ verbal behavior becomes highly relevant, especially when working with early age groups, as it contributes toward improving motivation, sports adherence, as well as promoting a number of disciplines, psychological and pedagogical dimensions (Keegan et al., 2009).

From an educational perspective, during the youth stages of sport development, coaches play a critical role in the teaching and learning processes as they are responsible for the player’s training, as well as their motivation to attend practice (Gilbert and Trudel, 2004).

Previous research has focused on the relevance of coaches’ intervention during the competitions (Partington and Cushion, 2012). Nevertheless, during the earlier formative stages, we consider the analysis of the pedagogical processes used during training sessions (typically developed during twice or three times per week) to be more relevant than the communication during the competition stage (once per week and with high contextual interference). The orientation of different teaching elements, such as the transmission of verbal information, will increase both the quality and the effectiveness of the training process. In this sense, findings by Hansen and Andersen (2014) have shown that coaches who express stimulating verbal behavior create a more effective learning environment guiding the athlete toward achieving their goals, as well as encouraging them to put more effort into training sessions. In this vein, Claxton (1988) demonstrated between-group differences in verbal communication of more successful and less successful coaches. In addition, López et al. (2016) highlighted that training using the Teaching Games for Understanding (TGfU), including the use of a questioning strategy, improves the motivation of the players and can have a positive effect on their performance. Furthermore, Zourbanos et al. (2007) found that positive feedbacks contributed toward encouraging a positive inner dialogue within players, while the opposite effect was observed when coaches used negative statements.

Coaches’ verbal behavior depends on many variables. Some studies have highlighted that the educational training represent the most relevant variables that influences the coaches’ verbal behavior (Leo et al., 2010; Huertas et al., 2018). On the other hand, the study carried out by Ford et al. (2010), points out the difference that may exist in the emission of verbal behavior by the coach depend on the context (match vs training) and the ages of the athletes. It is interesting to note that the study by Côté and Gilbert (2009) highlighted the importance of interpersonal relationships, the context where training takes place, the personality of the athletes and the results obtained by the team.

Studies have also analyzed the effect of coaches’ pedagogical intervention during competition (Leo et al., 2010; Grijalbo, 2015). For instance, Sánchez and Ramírez (2002) showed the importance of the coaches’ verbal behavior, highlighting that they generally provide information (predominantly tactical regardless of which team is winning/losing) to their players four times per minute, varying the information received by the player from between 8 and 36 messages per game. Similarly, Leo et al. (2010) reported that the coaches’ educational background is linked with their communication strategies during the competition, namely, showing that coaches with experience of university studies presented verbal interaction with greater means; using a more descriptive, prescriptive, and explanatory information and delving deeper into different specific contents of the activity than those coaches without university profile. However, it should be noted here that few studies have addressed the analysis of the coaches’ verbal behavior during the training process (Zetou et al., 2011; O’Connor et al., 2018), and at the time of writing, none have been focused on coaching at the grassroots training stages.

Regarding the importance of soccer coaches’ academic background, it is important to highlight that in a few countries (e.g., as Spain or Italy) obtaining the sports licenses for coaching in youth soccer can be achieved through several educational pathways (soccer federations, private authorized educational centers, or public centers regulated by the Ministry of Education, etc.). In addition, in Spain it is common for Sports Sciences University graduates not to enroll in coaching license courses during their university program. Previous findings have also revealed the differences in the academic content and time given to subjects related to Pedagogy and Training Methodology (Lledó, 2015). Consequently, it could be argued that coaches may employ different teaching methodologies depending on their educational background. It should be noted that coaches who have received a university degree in the subject area of Physical and Sports Education usually give more relevance to the general contents related to a holistic Physical Education perspective of sports training. Regarding this, Lledó et al. (2014) found that the Spanish University licensed coaches used a more comprehensive and participative methodologies, while coaches without a university background generally gave more importance to the sport-specific content using a more directive and less participative methodologies.

The focus of this research will be to analyze how the academic background of soccer coaches in Spain, specifically coaching younger age groups (U10 and U12), relates to the type and frequency of verbal behavior used during the training session. Given the different coaches’ educational background and variety of academic curriculum related with psycho-pedagogical and methodological content, we expect to find differences in the communication styles between coaches with different academic background. We hypothesized that the coaches with university experience will exhibit verbal behavioral patterns that are more suited to the requirements of young soccer players than coaches who lack a university level education.

## Materials and Methods

### Participants

The sample was composed of seventy youth soccer male coaches engaged in the training of U10 or U12 age categories in non-professional soccer clubs in the Region of Valencia (Spain). The coaches were aged from 20 to 35 years old (*M* = 25.87, SD = 4.00), with experiences of working as an Assistant or Head Coach between 2 and 10 years (4.53 ± 2.42). They were grouped according to their academic background in two different groups (see Table 1). One group was composed by 35 coaches with university studies (Diploma, Bachelor or Master’s Degree) related to Physical Education and or Sports Sciences (PESS), while the other group of 35 coaches did not have such a PESS university academic background. All coaches held the required license to train at these age groups. We selected exclusively male coaches to control the potential differences in communication and coaching styles between male and female coaches reported in several studies (Millard, 1996; Smucker and Whisenant, 2005; Bolter and Lucas, 2018).

**TABLE 1:** Characteristics of the coaches according to their academic background.

Coaching level	Academic background	Number of coaches	Age	Years of experience
Instructor	With university studies related with PESS	7	27.14 ± 2.41	7.00 ± 2.88
	Without university studies related with PESS	12	25.83 ± 5.06	3.75 ± 2.34
Level 1/UEFA B	With university studies related with PESS	18	25.77 ± 1.92	4.11 ± 1.74
	Without university studies related with PESS	16	23.37 ± 4.22	3.06 ± 1.38
Level 2/UEFA A	With university studies related with PESS	7	28.71 ± 4.42	6.71 ± 2.75
	Without university studies related with PESS	7	26.57 ± 4.85	4.85 ± 2.34
Level 3/UEFA PRO	With university studies related with PESS	3	28.33 ± 2.08	6.00 ± 1.73
	Without university studies related with PESS	0	0	0

We used this sample size without an *a priori* power analysis. Therefore, a sensitivity analysis was conducted using G^∗^Power (Faul et al., 2009) which showed that with our sample size (*N* = 70), the minimum effect size that could have been detected for α = 0.5, and 1 – β = 0.80, for two groups, is *f* = 0.33 (minimum detectable effect).

Participation in the study was voluntary and all of the coaches and club’s managers were briefed with regards to the general purposes of the study (analysis of the training methodology, without explicit reference to verbal behavior) prior to any data collection taking place. Selected coaches who met the inclusion criteria signed an informed written consent form. The study was conducted in accordance with the ethical standards of the 1964 Declaration of Helsinki (last update: Seoul, 2008) and was part of a larger research project that was approved by the Ethical Review Board of the Catholic University of Valencia under the reference UCV2015-2016-44-V.3.

### Procedure

Initially, researchers arranged an appointment with the managers of the clubs that met the inclusion criteria. During this first meeting, the goals of the study and its design were explained to the managers. Then after receiving each club’s approval, each coaches academic background was obtained from their curriculum vitae. Upon receiving all relevant documentation (parent and/or tutor informed consent), a schedule of the video recording for the training sessions was planned.

Data of the coaches verbal behavior was obtained using a video camera (*Nikon Coolpix A10*–Japan) and audio voice recording (*Wrist Band Bracelet*, Genius, Taiwan) during two training sessions of the same week during the same competitive period (January to May, 2017). The first training session was used to familiarize the coaches with the use of the bracelet, but the obtained data were not analyzed. In the second session, which took place on the last day of training prior to competition, we collected the data that would eventually be processed and analyzed. The initial transcription of the verbal behaviors, video and audio synchronization was performed through the *Microsoft Windows Movie Maker software*, version 5.1 (*Microsoft*, Redmond, WA, United States). After that, the verbal behaviors were coded in an *Excel spreadsheet* following the criteria described by Cushion et al. (2012) and Grijalbo (2015). Note that in the present study, we performed only the Step 1 of a modified version of the “*Coach Analysis and Intervention System*” (CAIS) (see Table 2) due to the reason that we analyzed the verbal behaviors used by the coach during the main part of the training (excluding warm up and cool down). Previous studies (Cushion et al., 2012; Stonebridge and Cushion, 2018; Eather et al., 2020) have confirmed the validity and reliability of CAIS in the context of study of verbal behavior in youth soccer.

**TABLE 2:** Definition of verbal behaviors according to the CAIS.

Verbal behavior	Definition
1. Positive modeling	Demonstrations, with or without verbal instruction, which shows the player the correct way to perform an action (example: demonstration of making correctly a short pass).
2. Negative modeling	Demonstrations, with or without verbal instruction, which shows the player the wrong way to perform an action (example: demonstration of making wrongly a long pass).
3. Physical assistance	Help the player to physically move their body to the correct position or through the correct movement trajectory (example: help the player to do a short pass moving his body in the correct way).
4 and 5. Specific feedback (positive or negative)	A specific verbal intervention (positive-supportive or negative-non-supportive) intended to offer information about the quality of the execution of an action (example: positive, “Good defense”; negative, “That attack was too slow”).
6 and 7. General feedback (positive or negative)	A general verbal intervention (positive-supportive or negative-non-supportive), which can be given during or after an action (example: positive, “Nice try”; negative, “Don’t try that again”).
8. Corrective feedback	An intervention containing information specifically aimed at improving a player’s execution of a future action. This can be provided simultaneously or at a later time (example: “Move further away from the goal when you attack,” “You need to speed up the movement of the ball”).
9. Instruction	Verbal signals that are reminders to instruct or direct the player behaviors related to their performance (example: “Speak up,” “Pressure,” “Keep them there”).
10. Humor	Jokes or comments to make the players laugh (example: “Are those steel cap shoes?”).
11. Hustle	Behavior who expressed intensity during the exercise (example: “Push more, push more, more intensity!”).
12. Praise	Positive or supportive comments that communicate the general satisfaction the coach feels toward one or several players. These do not always aim to improve their performance in the next action (example: “Good effort”).
13. Punishment	A specific punishment after a player commits an error (example: “Run 2 laps to the field for not training well”).
14. Scold	Negative comments or unsupportive comments that show disapproval toward a player and that are not specifically intended to improve their performance in the next action (example: insulting a player).
15. Others:	
a. Explanation/information	The coach will give the players the information regarding the training session (example: “The game will be a possession game, 8 vs 8 and the players will need to make 10 passes to score a goal”).
b. Alert/report	Verbal cues used to warn players about an event that is occurring at the same time or may occur at any moment (example: “Be careful, the defender is changing position”).
c. Encourage	Verbal comments or gestures that encourage or intensify a previously identified behavior (example: “You can do it, keep going” or “Come on”).
d. Game information/result	The information that the coach will relay will be to explain the aspects of the game, like the score or the time (example: “3 min left,” “2–0 to the blue team”).
e. Other uncategorized behaviors	It has not been heard or seen clearly. It does not belong to any other category.
16. Convergent and divergent questions	Convergent questions to the referee or another person about an action, strategy, procedure, score, or the wellbeing of a player. They only have one answer (example: “Are you okay?”). In contrast, divergent questions are those that do not have a single answer and invite bidirectionality, thus enhancing brain stimulation and creativity (example: “How do you think you can improve the shot on goal?”).
17. Response to question	Response to question which is not necessarily directly related to the competition (example: “Yes, you have to do it”).
18- Management criticisms	Management that is related to the match. Conduct to organize the team in terms of the game systems, the position of the players in the field, and the changes between them (example: “We will defend 5–1”, “Toni, defend in 3”).
19. Verbal protocol analysis	The coach thinks aloud or verbalizes thoughts and feelings (example: “yes, yes, I like it”).

### Data Analysis

Firstly, 30% of the verbal behaviors were analyzed and coded to determine the reliability and internal consistency of the CAIS model. The analysis of this process showed similar results to those obtained in previous studies (Cushion et al., 2012; Allan et al., 2016). Cohen’s Kappa index showed a value of 0.95 and was therefore deemed valid. The alpha reliability coefficient of Cronbach was 0.95, therefore it was considered reliable (Vaske et al., 2017).

Data analysis occurred in different steps. Initially, the frequencies, percentages, means, and standard deviations were calculated for each dependent variable, which were the 19 types of verbal behaviors analyzed from the CAIS, and for each coach. Finally, the one-way analysis of variance ANOVA were used for each of the categories defined by the CAIS to analyze the differences in the percentage of each type of verbal behaviors used by the coaches according to their academic experience. A level of significance of *p* < 0.05 was established. *F* and partial Eta-squared value were reported for the effect size. According to Richardson (2011) and Cohen (1969, pp. 278–280), benchmarks effect sizes were defined as small (0.0099), medium (0.0588) and large (0.1379). The SPSS version 24.0 statistical package was used for the data analysis.

## Results

A total of 32,886 verbal behaviors were registered, which were distributed according to the categories defined in the CAIS. Table 3 shows the distribution of the coaches’ verbal behaviors according to their academic experience.

**TABLE 3:** Percentage (±SD) of the distribution of the verbal behaviors of coaches according to their academic background.

Verbal behavior	Average	Non-university (%)	University (%)	*p*	*F*	$\eta_{p}^{2}$
Instruction	39.80 (8.88)	43.18 (9.07)	36.41 (7.37)	0.012**	6.60	0.08
Positive specific feedback	10.95 (5.40)	12.17 (6.19)	9.72 (4.22)	0.056	3.77	0.05
Hustle	7.20 (3.58)	6.50 (3.61)	7.89 (3.46)	0.957	0.00	0.00
Game information/result	5.67 (8.77)	7.04 (11.07)	4.30 (5.45)	0.150	2.12	0.03
Management direct	5.51 (3.69)	4.75 (3.52)	6.26 (3.74)	0.184	1.80	0.02
Other uncategorized behaviors	4.62 (2.71)	4.45 (2.99)	4.78 (2.41)	0.201	1.66	0.02
Encourage	4.48 (2.94)	4.70 (3.06)	4.26 (2.83)	0.576	0.31	0.00
Negative general feedback	3.63 (2.43)	2.73 (2.02)	4.53 (2.49)	0.008*	7.48	0.09
Positive general feedback	2.90 (2.61)	1.99 (2.04)	3.80 (2.82)	0.026*	5.18	0.07
Response to question	2.77 (1.81)	2.38 (1.46)	3.15 (2.05)	0.188	1.77	0.02
Explanation/information	2.61 (1.24)	2.30 (1.26)	2.91 (1.15)	0.080	3.16	0.04
Corrective feedback	2.44 (1.74)	1.84 (1.35)	3.04 (1.89)	0.018*	5.86	0.07
Negative specific feedback	1.96 (2.09)	1.58 (1.58)	2.33 (2.46)	0.305	0.30	0.01
Alert/report	1.62 (2.12)	1.31 (1.94)	1.92 (2.26)	0.707	0.14	0.00
Convergent question	1.31 (1.18)	0.84 (0.90)	1.77 (1.26)	0.002*	9.96	0.12
Divergent question	0.75 (0.88)	0.58 (0.83)	0.92 (0.91)	0.159	2.02	0.02
Humor	0.72 (1.44)	0.26 (0.45)	1.18 (1.90)	0.003*	9.33	0.12
Positive modeling	0.39 (0.45)	0.33 (0.34)	0.45 (0.54)	0.360	0.84	0.01
Verbal protocol analysis	0.38 (0.40)	0.42 (0.41)	0.33 (0.39)	0.319	1.00	0.01
Scold	0.32 (5.55)	0.17 (0.22)	0.47 (0.73)	0.060	3.67	0.05
Praise	0.26 (0.51)	0.22 (0.25)	0.30 (0.69)	0.874	0.02	0.00
Negative modeling	0.07 (0.17)	0.05 (0.11)	0.09 (0.22)	0.494	0.47	0.00
Punishment	0.05 (0.15)	0.07 (0.18)	0.03 (0.11)	0.119	2.49	0.03
Physical assistance	0.00	0.00	0.00	–	–	–
Total	100	100	100	–		

*Significant statistically differences in favor of coaches with university studies. **Significant statistically differences in favor of coaches without university studies.

**FIGURE 1:**
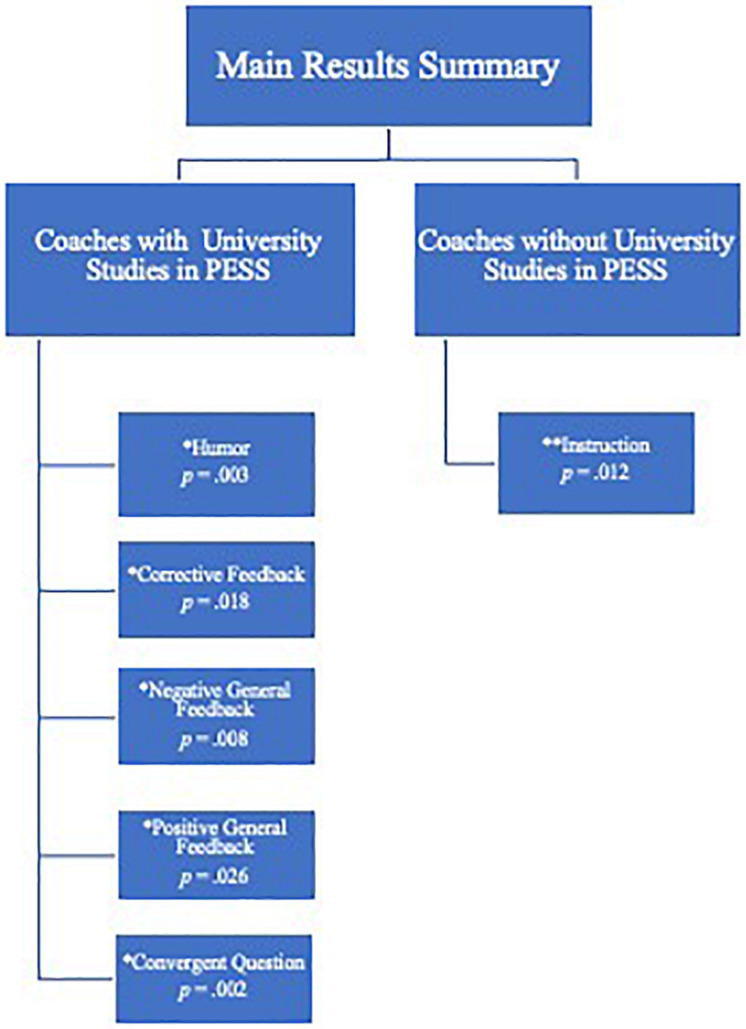
Main results summary. *Verbal behaviors with significant differences in favor of Coaches with university studies in PESS. **Verbal behaviors with significant differences in favor of Coaches without university studies in PESS.

Our results (Figure 1) showed significant differences between the coaches according to their academic background, observing that coaches with an university degree in PESS used 1.8% more negative general feedback [(*F*(1,68) = 7.48, *p* = 0.008, $\eta_{p}^{2}=0.09$)], 1.81% positive general feedback [(*F*(1,68) = 5.18, *p* = 0.026, $\eta_{p}^{2}=0.07$)]; 1.2% corrective feedback [(*F*(1,68) = 5.86, *p* = 0.018, $\eta_{p}^{2}=0.07$)], 0.93% convergent question [(*F*(1,68) = 9.96, *p* = 0.002, $\eta_{p}^{2}=0.12$)], and 0.92% more humor [(*F*(1,68) = 9.33, *p* = 0.003, $\eta_{p}^{2}=0.12$)] than coaches without university studies in PESS.

In contrast, the coaches without university studies related with PESS used 6.77% more instruction than those who did have that academic education [(*F*(1,68) = 6.60, *p* = 0.012, $\eta_{p}^{2}=0.08$)].

There were no significant differences between the groups of coaches in the following behaviors: positive specific feedback (*p* = 0.56), hustle (*p* = 0.957), game information/result (*p* = 0.150), management criticisms (*p* = 0.184), others (*p* = 0.201), encourage (*p* = 0.576), response to question (*p* = 0.188), negative specific feedback (*p* = 0.305), alert/report (*p* = 0.707), divergent question (*p* = 0.159), positive modeling (*p* = 0.360), verbal protocol analysis (*p* = 0.319), praise (*p* = 0.874), negative modeling (*p* = 0.494), scold (*p* = 0.060), explanation/information (*p* = 0.080), and punishment (*p* = 0.119). Physical assistance behavior was not observed in either group.

## Discussion

The main objective of this study was to analyze the relationship between the coaches’ verbal behavior and academic background in PESS. The present study is novel in approaching the study of coaches’ verbal behavior in relation to their academic experience. Earlier research by Lledó et al. (2014) observed that coaches with an experience of university PESS studies conferred greater importance to the use of inclusive methods of coaching delivery, while those who did not have PESS university experience rated more directive methods more positively. It should be noted, however, that the details relating to these preferences were gained through self-reporting and there was no direct observation of the methods and teaching approaches used by the coaches.

The results obtained in our research have allowed us to confirm the stated hypotheses. In general, our results showed that those coaches who have completed university studies related to PESS showed more varied verbal behavior patterns related to the pedagogical and methodological needs of young players in categories U10 and U12 than those coaches without PESS university experience. Similar to other previous studies, the coaches in this study with PESS University used positive behaviors more frequently in their coaching. These included feedback behaviors (Wulf et al., 1998, 2002), verbal questioning behaviors (Averill et al., 2016; Theeboom et al., 2016), and humor (Spencer, 1996; Banas et al., 2019). Our results have shown that the coaches with PESS university studies used more general feedback, both negative and positive, than coaches without. The use of this type of general feedback, especially positive feedback, has been deemed useful to create positive learning environments (Mouratidis et al., 2008). Also, the use of positive feedback is in line with the long-term training principles described by Jayanthi et al. (2013) and Kliethermes et al. (2020), as well as, it is noted in the soccer related studies by Ford and Williams (2012) and Haugaasen et al. (2014). Studies carried out in other sports have also shown the importance of feedback. In basketball and volleyball, Iglesias et al. (2007) and Markland and Martinek (1988), respectively highlighted the positive influence of positive verbal behaviors on strengthening the self-esteem and self-confidence of the players. In addition, it helped their level of involvement in the game and influencing an improvement in the intuitive decisions making of the players.

Furthermore, our results showed that the coaches with university studies frequently used more corrective feedback, convergent and divergent questions, which are important to create pedagogical environments centered on the athletes’ development. A study by Tzetzis et al. (2008) showed that the proper use of corrective feedback in the teaching of skills of different levels of difficulty is useful in empowering young athletes and the enhancement of their self-confidence.

Concerning the use of questions, the greater use of convergent and divergent questions by coaches with a PESS university experience may stimulate an athletes’ autonomy and intrinsic motivation (Moen, 2012). Both types of questions have an essential role in any child’s cognitive development, especially divergent questions. These are characterized by having an unlimited number of responses, the seeking of, and, exchange of opinions and requiring a high level of thinking that will favor a child’s cognitive restructuring process (Wittmer and Honig, 1991; Felicia, 2019). Through its implementation, it may be possible to optimize a higher level decision making of the player (Harvey and Light, 2015). Thus, promoting divergent thinking within an individual is deemed a key aspect in the long-term performance of soccer players (Memmert et al., 2013).

Our results are in line with those outlined by Lledó and Huertas (2012) and Lledó et al. (2014), verifying that coaches with the experience of PESS university studies valued the use of more inclusive teaching styles in their training, such as guided discovery and problem-solving, where converging and diverging questioning are more frequently used. In contrast, the coaches without university experiences in PESS rated the use of more traditional-managerial models more positively than coaches with university experiences.

It is also important to note that the coaches with PESS university studies used humor behaviors to a greater extent than those without. This type of behavior will favor the optimization of positive teaching-learning environments (Halula, 2013; Hu et al., 2017) which may improve the mood and influence the emotional status of the players (Ronglan and Aggerholm, 2014; Grijalbo, 2015).

For the coaches without university studies in PESS, it should be highlighted that they used more instructions than the PESS coaches. Instruction is considered as a verbal behavior associated with the “directive behaviors.” This type of verbal behavior limits the development of autonomy and creative thinking in young people, significant perspective for improving medium- and long-term performance of soccer players (Light and Harvey, 2017). These types of verbal behaviors are more typically found in directive learning styles, which do not necessarily respond to the cognitive, adaptive, and creative needs of the learning process (Morgan et al., 2005). In general, our findings are in line with those described by Lledó (2015) and Leo et al. (2010), showing that the coaches without PESS experience valued directive instructional strategies more than those coaches who have completed PESS university studies.

Concerning the limitations of our study, it is important to note that despite the high number of verbal behaviors coded (32,886), the effect sizes obtained when comparing both groups and obtaining significant differences were medium (from than 0.07 to 0.20). This could be explained by the variability between participants, due to the coaches’ verbal behavior during the only one training session analyzed. This, in turn, could have been influenced by different contextual variables affecting the team (e.g., daily training goals, result of previous matches, characteristics of the next match, classification of the team, new players in the group, …) or external personal problems affecting the coach. Although we consider that this variables are not affecting the main result in our study because they could affect both groups of coaches similarly, our findings should be cautiously interpreted. Future extended follow-up studies during a longer time might consider measuring the influence of these variables on the coaches’ behavior and more importantly, how the players develop their general and specific technical, tactical, physical, and psychological skills effectively. Other limitations in the present research is the exclusion of Spanish professional clubs (LaLiga Santander and LaLiga SmartBank Clubs) due to the fact that previous studies have shown that most coaches in the academies of professional clubs are already university graduates (Lledó, 2015), which would generate a bias when analyzing and interpreting the results. On the other hand, the conclusions of the present study cannot be extrapolated to female football as no female teams or female coaches were included in the selected sample. Further research should include and compare the verbal behavior used by female coaches. Finally, it should be noted that the outcomes of our investigation cannot be fully generalized based on the diversity of the educational coaching structure in different countries.

## Conclusion

Our findings allow us to affirm that the coaches with the experience of PESS university academic studies use verbal behaviors that could stimulate the athlete’s active participation and cognitive development. Conversely, those coaches who have not obtained a university education may tend to use, to a greater extent, a more directive and less participative instructive behaviors.

Since it has been shown that the coaches academic background modulates the quality of the communication processes, we propose the review of the coaches’ educational curriculum to promote a greater understanding of the pedagogical aspects related to coach-athlete communication. A step forward in the coaches education process, that we believe, will impact positively in the sporting development of young people.

## Data Availability Statement

The raw data supporting the conclusions of this article will be made available by the authors, without undue reservation.

## Ethics Statement

The studies involving human participants were reviewed and approved by the Ethical Review Board of the Catholic University of Valencia under the reference UCV2015-2016-44-V.3. Written informed consent to participate in this study was provided by the participants’ legal guardian/next of kin.

## Author Contributions

DA: conception and design of the study, data acquisition, statistical analysis and interpretation, drafting of the article, final approval of the version to be published, and agreement to be accountable for all aspects of the research. RB and WT contributed to data interpretation, critical revision of the study, final approval of the version to be published, and agreement to be accountable for all aspects of the research. JJ-B: data acquisition and interpretation, final approval of the version to be published, and agreement to be accountable for all aspects of the research. FH: main role in the conception and design of the study with a relevant role in project coordination, statistical analysis and interpretation, critical revision of the article, and final approval of the version to be published, and agreement to be accountable for all aspects of the research. All authors contributed to the article and approved the submitted version.

## Conflict of Interest

The authors declare that the research was conducted in the absence of any commercial or financial relationships that could be construed as a potential conflict of interest.
